# Experimental investigation of a solar absorber and nocturnal radiator hybrid system for simultaneous water heating and cooling

**DOI:** 10.1016/j.heliyon.2021.e07989

**Published:** 2021-09-14

**Authors:** Kago R. Rabasoma, Aobakwe Kolobeng, Kevin N. Nwaigwe

**Affiliations:** Department of Mechanical Engineering, University of Botswana, Gaborone, Botswana

**Keywords:** Nocturnal cooling, Diurnal heating, Hybrid, Thermal performance, Temperature

## Abstract

An experimental study of a Solar Absorber and Nocturnal Radiator (SAANR) hybrid system used for simultaneous water heating and cooling in Gaborone, Botswana is presented. A SAANR hybrid system was designed and fabricated and the system consists of major components that include a 1.4 m^2^ SAANR hybrid panel, 100 L hot and cool water storage tanks, chlorinated polyvinyl chloride (CPVC) plumbing and a 0.2778 kg/s circulation pump, all mounted on a steel frame that is inclined at 30 °C to maximize solar absorption and self-cleaning of the panel. Experiments carried out in December 2020 in Gaborone with water at an initial temperature of 26 °C produced 100 L of hot water at 60 °C and similarly 100 L of cool water at 23 °C, during the day and at night respectively. These results provide proof that the SAANR hybrid system could be used simultaneously for applications of domestic water heating and building envelope cooling in Botswana and similar climates. This technology can therefore be used instead of electric water heaters and air-conditioners and thus save energy.

## 1 Introduction

It is widely reported that nocturnal radiation cooling is one of the most effective natural passive cooling technologies by infrared radiation exchange between earthly surfaces and the sky [1, 2, 3, 4, 5, 6, 7, 8, 9, 10, 11]. The sun radiates heat to the earth and warms it up during the day, but at night the warmer earth would radiate the heat to the then colder sky. These processes can be harnessed for solar collection and nocturnal radiation cooling applications respectively [12, 13, 14, 15, 16, 17, 18, 19, 20, 21, 22, 23, 24, 25]. Combining both solar energy collection and nocturnal radiative cooling processes in a single hybrid system would significantly reduce investment costs for heating and cooling as compared to having standalone heating and cooling systems [12, 26]. For a hybrid system, the maximization of solar absorption directly counteracts night-time radiation properties, hence a balance has to be achieved to serve both functions of the hybrid system by forgoing peak performance in both the heating and cooling loops. This balance takes into consideration the intended application of the system as well as the minimum needs for cooling and heating in the climatic region [26].

Several researchers have studied the application of hybrid systems in different climates and using many different approaches. In a study investigating five different locations in Nigeria, and utilizing finite element numerical analysis, authors achieved maximum temperatures of 84.6 °C, 75.6 °C, 86.4 °C, 88.3 °C, and 93.7 °C in the heating phase, and minimum temperatures of 20.2 °C, 20.1 °C, 21.9 °C, 20.9 °C and 22.0 °C in the cooling phase for the different locations respectively [12]. In another study, Balen, et al. [26] conducted an extensive analysis of flat plate radiative panels operation when integrated into the space ventilation system for a maritime climate in Ireland. The panels were designed with the primary function of producing sufficient quantities of cold water by integrating night-time radiative and convective cooling, however, the panels were modified during the day to operate as solar collectors and to be used to produce hot water. The study developed a simulation model for the parametric analysis of the system in summer operating conditions, from which results yielded a water temperature drop of 12 °C in night-time operation and a temperature rise of 35 °C during daytime heating at a 67-degree tilt angle. Some studies have targeted investigating the benefits of controlling passive energy systems, such as the work of Parsons and Sharp [27] which studied the benefits of using several arrays of heating and cooling systems in buildings in Louisville, USA. Findings from the research showed that using a combined system of passive heating and cooling has energy-saving benefits, but even bigger energy savings were attained by actively controlling passive systems. The best-performing systems were shown to be those that have a control strategy that changes the cooling/heating mode in response to forecasted temperature abnormalities that occur during the season [27].

Recently, Rabasoma and Nwaigwe [28] studied analytically the application of a hybrid panel in a subtropical location of Gaborone, Botswana. The study developed mathematical models for a solar absorber and nocturnal radiator hybrid panel operating in the summer and winter seasons of Botswana using a finite difference method. Transient analysis and performance prediction of the hybrid panel for water heating and cooling were undertaken and the hybrid panel simulations produced cold water of 22.8 °C minimum temperature and hot water of 49 °C maximum temperature. These results showed the possible efficacy of solar absorption and nocturnal radiation hybrid systems in Southern African semi-arid climates, however, there still existed a research gap in that the studies were theoretical without experimental backing. The current work is an extension investigating experimentally, the performance of a typical hybrid panel within the study area. This study corroborates the numerical studies carried out earlier and develop optimal experimental tests for use of hybrid panels in subtropical regions. A novel manually switchable hybrid system was designed, fabricated and tested, which included among its major components, a glazed solar collector and nocturnal radiator (SAANR) hybrid panel that is simple to fabricate and yet also a very robust design that can be scaled up as well as improved upon by material adjustments. These properties render the hybrid system under study favourable for application in the industry, along with ease of integration to already existing installations.

## 2 Methodology

**Figure 1:**
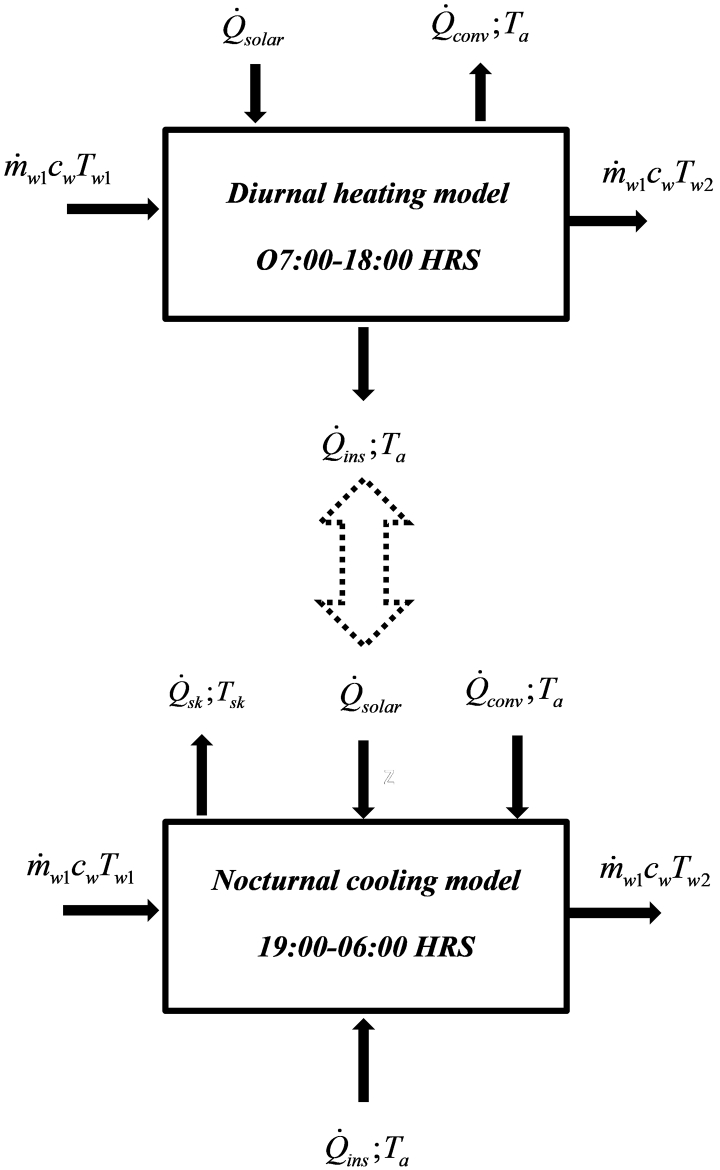
Processes summary in a hybrid solar absorber and nocturnal radiator.

For the experimentation, the panel was designed to operate in two phases, the heating phase during the day and the cooling phase at night as shown in Figure 1.

### 2.1 Experimental setup and procedure

**Figure 2:**
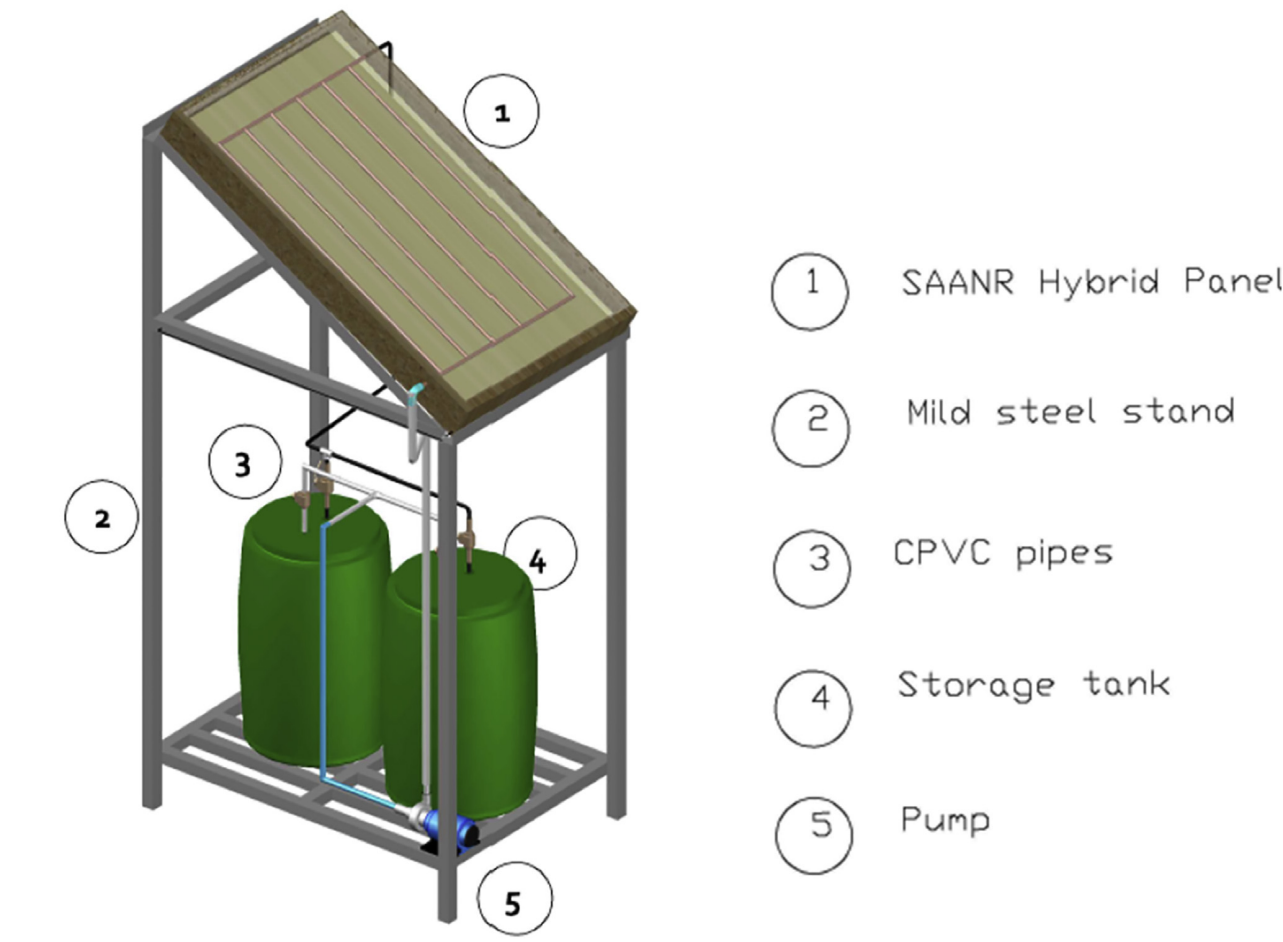
SAANR experimental setup.

**Figure 3:**
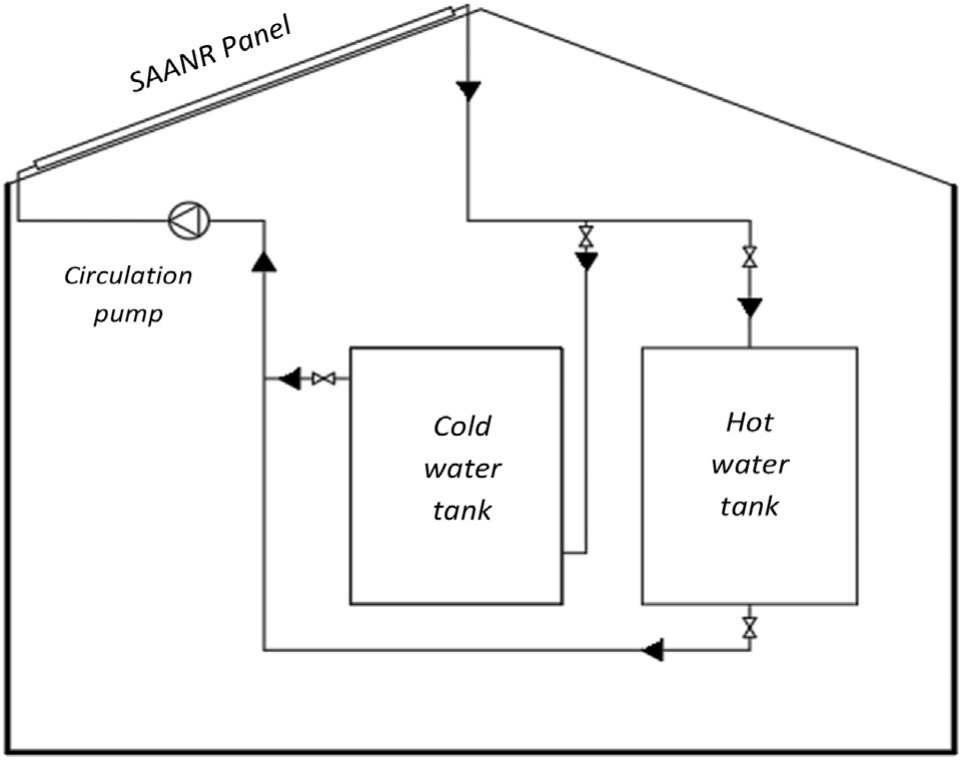
Hydraulic Illustration of the Experimental rig.

The experimental setup (Figure 2) was designed and fabricated to investigate a SAANR hybrid system in Gaborone, Botswana. The system consists mainly a 1.4 m^2^ SAANR hybrid panel, two 100 L storage tanks for hot water and cool water, a 0.2778 kg/s constant flow circulation pump, connection plumbing made of chlorinated polyvinyl chloride (CPVC) which is suitable for both hot and cold-water operation and a steel frame to support the setup. The storage tanks and CPVC piping were all insulated to minimize heat transfer energy losses in the experiment. The hydraulic connection of the experiment (Figure 3), shows the schematics of the major components of the system looped together, including safety devices.

**Figure 4:**
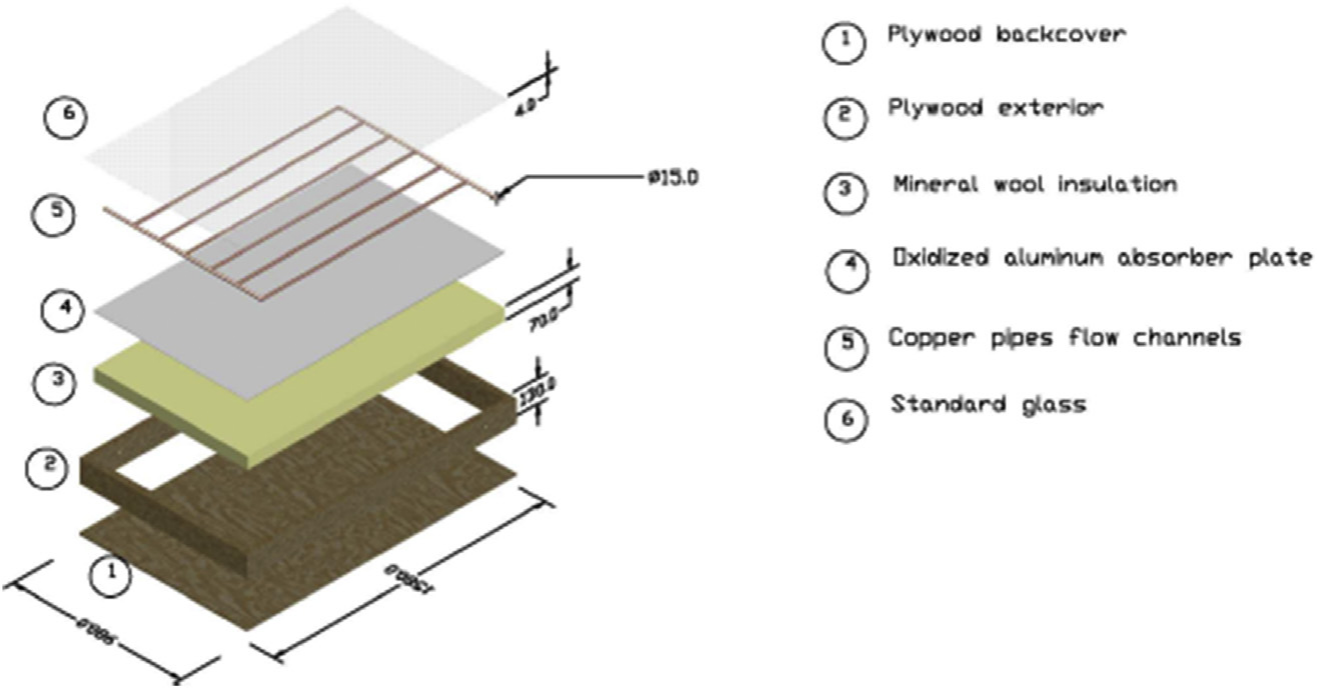
SAANR hybrid panel.

**Figure 5:**
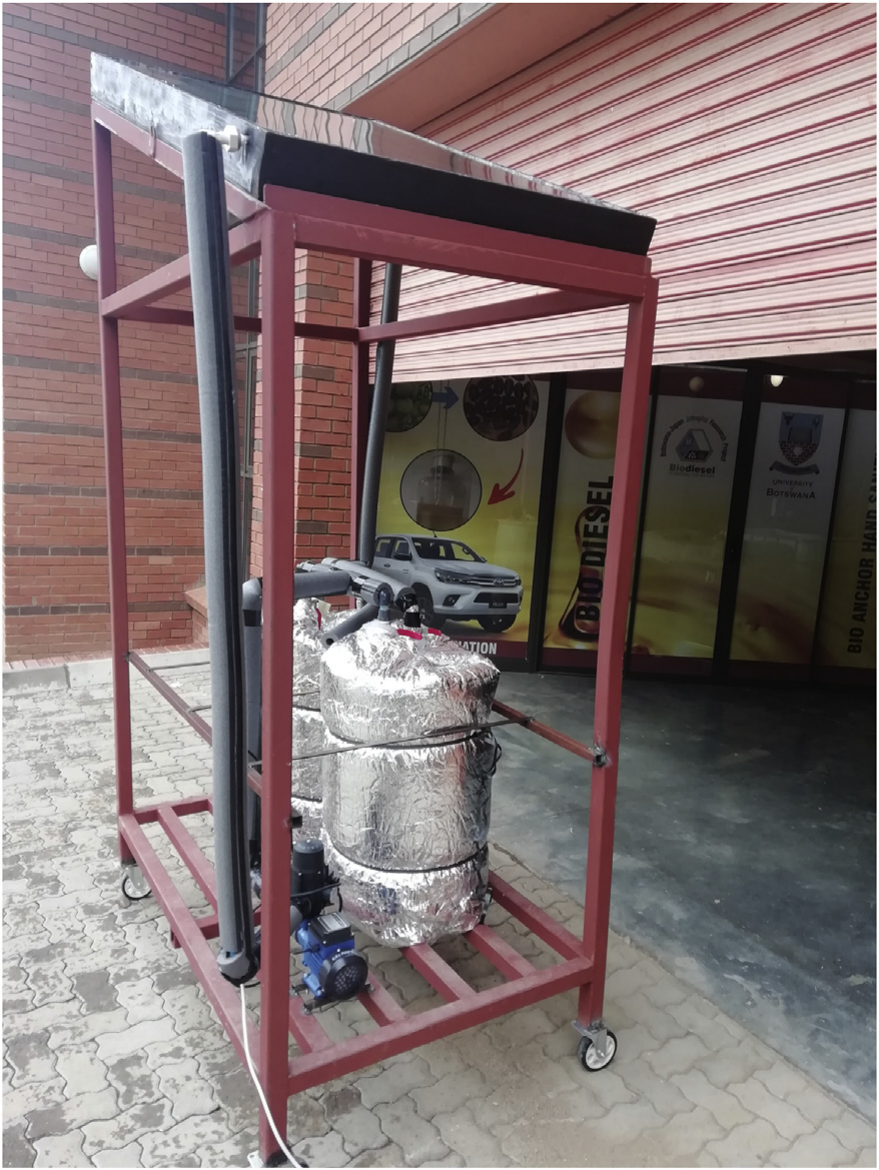
SAANR hybrid system in operation at the University of Botswana.

The SAANR hybrid panel shown in Figure 4 is made up of plywood on the exterior, with an oxidized aluminium thin absorber plate which is reasonably favourable for both solar absorption and night-time radiation. On top of the absorber plate, 15 mm copper tubes were bound in a harp arrangement, and the whole panel was covered by regular glass to reduce convection effects. A thick layer of mineral wool was used to insulate underneath the absorber plate to ensure minimal heat transfer through the back of the panel casing. The surface area of this panel is 1.4 m^2^. Steel square pillars were used to fabricate the frame. The frame has an inclination angle of 30° which is relatively close to the local latitude of 24° to maximize solar absorption [29], as well as have enough inclination to achieve self-cleaning of the panel. The heating experiment was run from 7 am to 6 pm, and then the experiment was switched to run the cooling loop experiment through the night from 7 pm until 6 am of the following day. The output of the experiment is hot water during the day and cool water at night, produced through solar absorption and nocturnal radiation respectively, and stored in their respective storage tanks. Results of water temperature from the Hot water tank and the Cold-water tank were recorded at hourly intervals using a data logger for a 24-hour day. Figure 5 shows the complete SAANR hybrid system experimental test rig in operation at the University of Botswana Engineering Projects Lab.

## 3 Results and discussion

**Figure 6:**
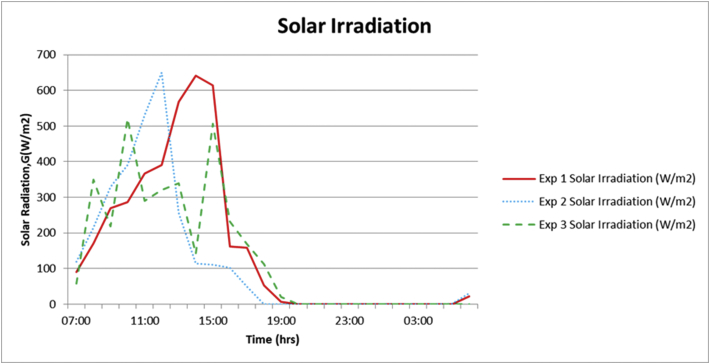
Global solar irradiation during experiment trials.

The output from the experiments was hot water and cool water stored in their respective tanks. The temperatures in the two tanks were captured at hourly intervals. A total of 3 experiments were recorded, experiment 1 was from the 3^rd^ to the 4^th^ of December 2020 and the weather was partly cloudy. Experiment 2 was carried out from the 9^th^ to the 10^th^ of December 2020 where the weather was partly cloudy with some intermittent rain showers in the afternoon and evening. Experiment 3 was on the 14-15^th^ December 2020 in partly cloudy weather conditions. Solar radiation has the greatest influence on the performance of the heating loop, thus the output of the system was assessed relative to the solar radiation on the referenced date. Hence, irradiation levels on the dates of the experiments were measured and recorded as shown in Figure 6. The irradiation levels during these experiments reached up to 600 W/m^2^ which is relatively below expectation, considering that this was during a summer month. December 2020 in Gaborone has had more rains than usual and was partly cloudy on most days thus the low solar radiation levels.

**Figure 7:**
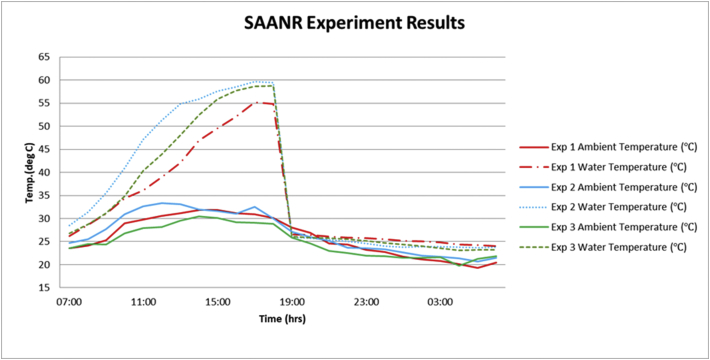
SAANR hybrid system experiment results.

The experimental results of the hybrid system (Figure 7) produced hot water up to a maximum of 60 °C, translating to a temperature rise of about 34 °C from the initial temperature of 26 °C. Experiment 1 performed the poorest for water heating amongst all the experiments, but even then, it was able to produce hot water at 55 °C. The hot water produced indicates that the system can be applied for solar water heating in Botswana taking into consideration the ideal domestic hot water temperatures reported by Carlos, et al. [30]. The cooling loop experiments produced cool water at a minimum of 23 °C, with experiment 3 performing the best. This represents a 3 °C temperature drop during the nocturnal cooling loop. This result compares well with earlier results by other researchers [23, 31, 32]. Cool water can be applied for in-room cooling with the aid of water to air heat exchangers especially in the summer months of Botswana where ambient day temperatures are usually well above 30 °C. In nocturnal cooling, the cooled space or medium serves as the heat source while the sky serves as the heat sink. The temperature of the heat source tends towards the temperature of the heat sink which is usually lower. In a situation where the temperature of the cooled space/medium is lower than the ambient temperature, convective heat transfer is set up from the ambient to the cooled space/medium, and this heat gain is radiated to the sky, hence maintaining nocturnal cooling on the cooled space/medium. In the present experiment, convective heat transfer did not take place during the nocturnal loop as ambient temperature was lower than the temperature of the cooled space. To prevent convective heat transfer between the ambient and a cooled space/medium, the attic is usually designed to insulate the system from undergoing a reversed situation or temperature sensors are used to stop circulation for other applications [33].

**Figure 8:**
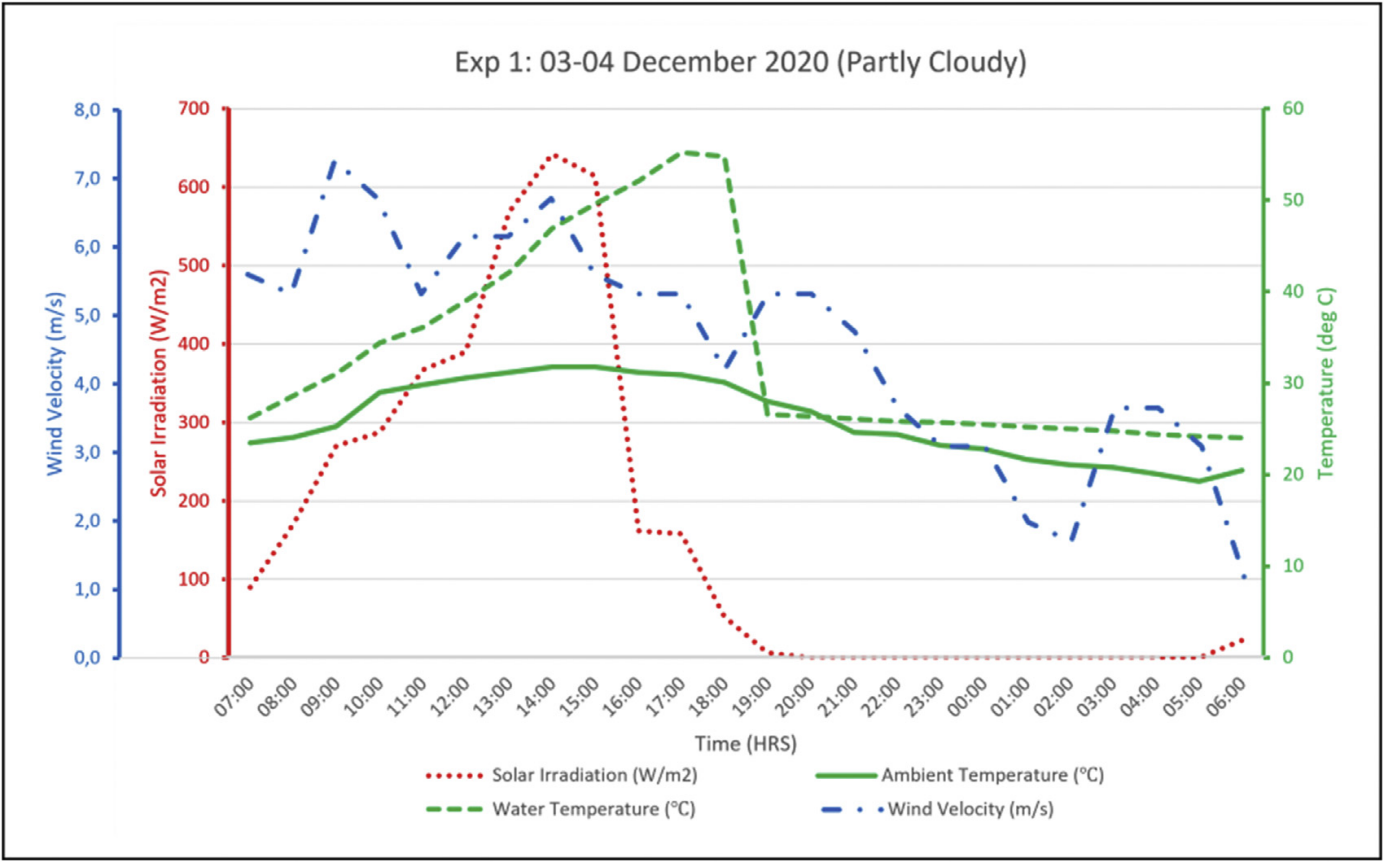
Experiment 1 results.

**Figure 9:**
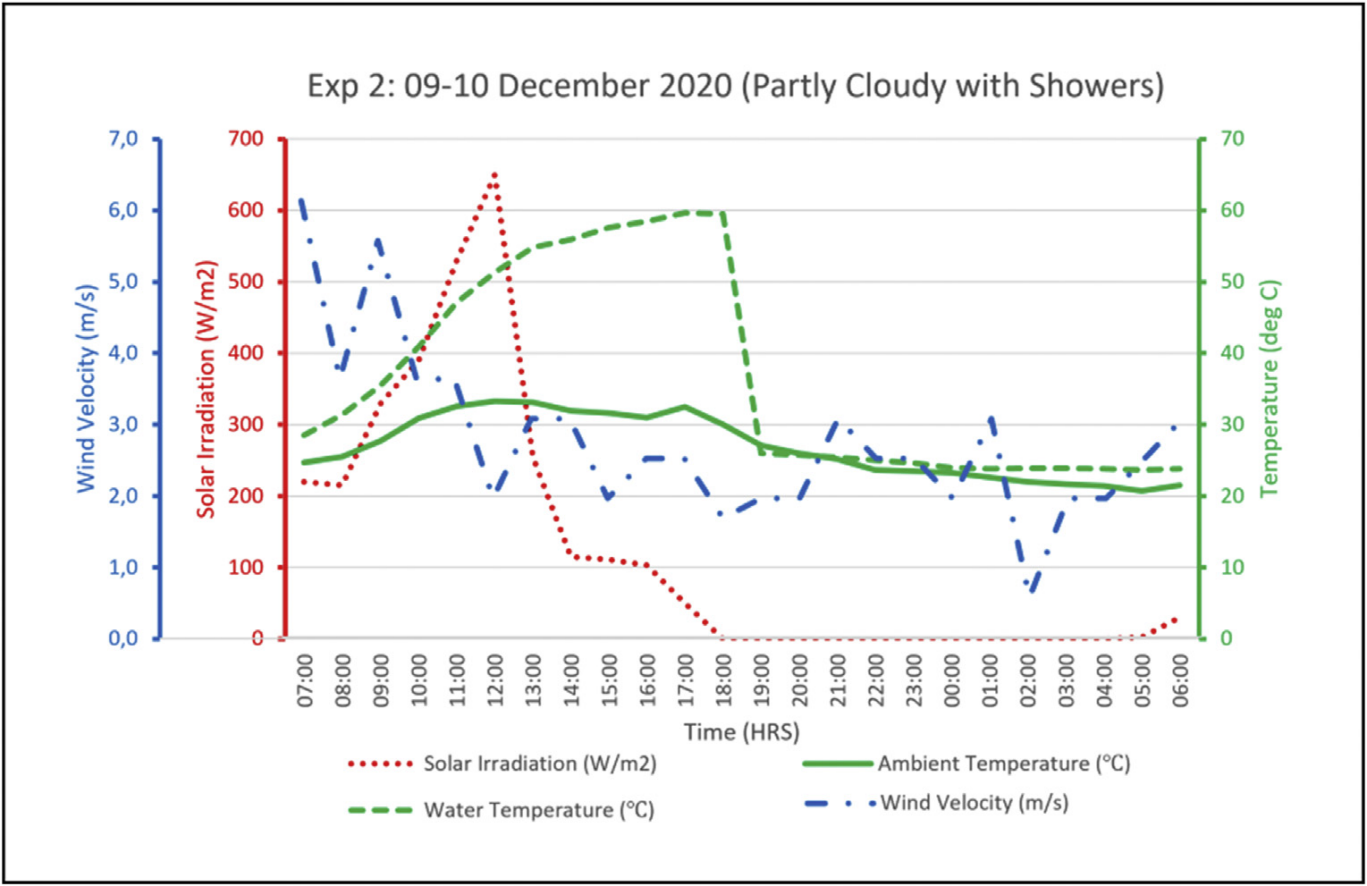
Experiment 2 results.

**Figure 10:**
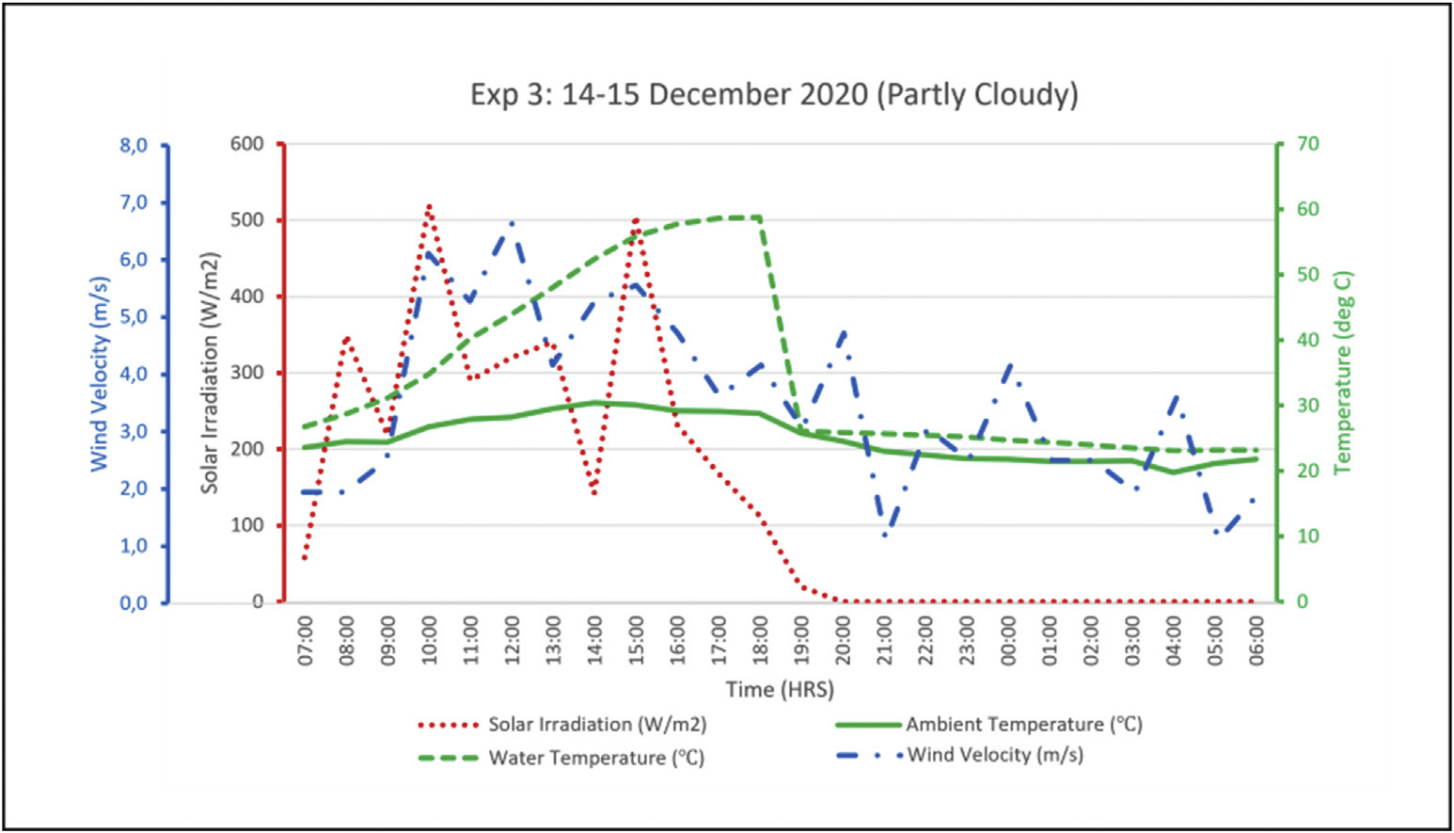
Experiment 3 results.

The following figures (Figures Figure 8, Figure 9, Figure 10) present the experimental output alongside its associated parameters. From the aforementioned figures it is shown that hot water temperature is directly influenced by the levels of solar radiation, as the trend of outlet water temperature generally followed after the trend of solar radiation. The water temperature curve even flattened as solar irradiation diminished in the afternoons. A similar observation was made in linking ambient air temperature with the outlet water temperature. Night-time radiation cooling is most effective on a clear sky as reported by several researchers including Okoronkwo, et al. [11] as does solar water heating. So, the experiments being carried out on partly cloudy weather conditions would have certainly affected performance of the experimental rig on both the cooling and heating loops. Wind velocities throughout the experiments ranged between 3.0-6.0 m/s (as shown in Figures 8–10) which is fairly normal for a typical summer season in Botswana. However, the strong winds might have reduced the cooling capacity of the hybrid system taking into consideration the reports from studies by Yoshihiro and Masato [34] that strong ambient winds suppresses or delays nocturnal cooling. Another aspect that might have diminished the cooling capacity of these experiments is the high relative humidity. As reported earlier, December 2020 was blessed with regular rainfall which consequently increased the relative humidity. Humid environments hinders the nocturnal radiation cooling process [13].

## 4 Conclusions

A Solar Absorber and Nocturnal Radiator (SAANR) hybrid system was designed, fabricated, and tested in Gaborone, Botswana. Experiments of the SAANR hybrid system using 100 L of water for both the hot water tank and cool water tank showed temperature gain of up to 34 °C for the hot water storage tank and cooling of up to 3 °C in the cold-water storage tank. The best performing experiment trials produced 60 °C hot water, and also produced cool water at 23 °C daily in each storage tank, hence the experimental rig has proven the viability of a hybrid solar absorber and nocturnal radiator system for use in producing domestic hot water and cool water in Botswana climate. The cool water can be used in various applications of building envelope conditioning instead of regular grid electricity-powered air-conditioners. It is also concluded from this study that the performance of the hybrid system can further be improved by substituting with specialist materials especially for the absorber/radiator plate, and also by increasing the size of the panel or connecting several SAANR panels in series to improve performance and parallel to increase the yield.

## Declarations

### Author contribution statement

Kago R. Rabasoma: Conceived and designed the experiments; Performed the experiments; Analyzed and interpreted the data; Contributed reagents, materials, analysis tools or data; Wrote the paper.

Aobakwe Kolobeng: Performed the experiments; Contributed reagents, materials, analysis tools or data; Wrote the paper.

Kevin N. Nwaigwe: Conceived and designed the experiments; Analyzed and interpreted the data; Contributed reagents, materials, analysis tools or data; Wrote the paper.

### Funding statement

This work was supported by AEE INTEC Austria through the SOLTRAIN IV graduate student bursary award.

### Data availability statement

Data included in article/supp. material/referenced in article.

### Declaration of interests statement

The authors declare no conflict of interest.

### Additional information

No additional information is available for this paper.
